# Do ward rounds offer effective teaching and training? Obstacles to learning and what makes good teaching in a large tertiary care hospital from trainee doctor’s perspective

**DOI:** 10.3205/zma001502

**Published:** 2021-09-15

**Authors:** Mohammad Ayaz Khan, Rajkumar Rajendram, Hamdan Al-Jahdali, Abdullah Al-Harbi, Majed Al-Ghamdi, Imad Hasan, Mostafa Mohammad Obaidi, Emad Masuadi

**Affiliations:** 1 King Saud University for Health Sciences, College of Medicine, Riyadh, Saudi Arabia; 2 Ministry of National Guard-Health Affairs, Department of Medicine, Pulmonary Division, Riyadh, Saudi Arabia; 3 King Abdullah International Medical Research Centre, Riyadh, Saudi Arabia; 4 Ministry of National Guard-Health Affairs, Department of Medicine, Internal Medicine Division, Riyadh, Saudi Arabia

**Keywords:** ward round, training, medical education, perception

## Abstract

**Background: **Ward rounds (WR) have been integral to the process of teaching and learning medicine and also provides a vital opportunity to communicate with the patient, their relatives, and other healthcare professionals. Yet in recent years trainees’ perception of the educational value of WRs seems to have declined.

**Objectives: **The aim of this study to assess trainees’ perception of the educational value of WRs at King Abdulaziz Medical City(KAMC), Riyadh, a 1500 bed academic hospital in Saudi Arabia.

**Methods:** A self-administered, paper-based survey was distributed to physicians in training at KAMC between October and December 2019. All residents who attended WRs were invited to participate. The questionnaire was adapted from a survey used in a previous study. The demographic section requested details of the respondent's age, gender, specialty, and seniority. The second and third sections asked about the logistics of current ward round practices. It included several questions on the structure as well as the duration and frequency of ward rounds. The fourth and fifth sections asked for participant’s perception of the opportunities for, and the obstacles to, learning on ward rounds. The subsequent sections asked several questions onward round structure and the clinical teacher. Responses were requested on a 5-point Likert-type scale (strongly disagree, disagree, neutral, agree, strongly agree). The last section asked the participant for general comments and feedback

**Result: **The study targeted 250 residents in specialties that routinely performed WRs. Only 166 residents returned the questionnaire (response rate of 66.4%). Male 89 (53.6%), medical 108 (65.1%), surgical 58 (34.9%), resident in first year 81 (48.8%). The overall average time spent on WR was 13 (± 11 SD) hours per week. The WR was perceived as a good opportunity to learn about diagnostic investigation 138 (83%) and patient management 133(80.1%), history taking114 (68.7%) physical examination 103 (62.0%), and time management skills 86 (51.8%). The majority of our trainees felt that the WR was educationally very useful to 86 (52%) and attribute to at least a third of the education they receive during their training. They also reported that about the quarter of the time spent on WRs is devoted to teaching. The good teacher described as enthusiastic to teach 137 (82.5%), provide feedback to trainees 135 (81%), do not rush 139(83.7), communicate to trainee 144 (86.7), and consultant level,101 (60.8). Trainees also identify a few factors that hinder their training such as lack of time 130 (79%), and the number of patients 129 (78.3).

**Conclusion: **This study identifies the strengths and weaknesses of WR in our institution. Finding will help training supervisors in addressing and rectifying these shortcoming and factors hinder training.

## Zusammenfassung

**Hintergrund: **Visiten sind ein integrativer Bestandteil des Lehrprozesses und des Lernens in der Medizin und bieten zudem eine sehr wichtige Möglichkeit, um mit Patient*innen, deren Verwandten und anderem Gesundheitspersonal zu kommunizieren. Und trotzdem scheint in den letzten Jahren der Ausbildungswert von Visiten in der Wahrnehmung der Ärzt*innen in der Ausbildung nachzulassen.

**Ziele: **Ziel dieser Studie ist es, die Wahrnehmung der Ärzt*innen in der Ausbildung hinsichtlich des Ausbildungswertes von Visiten in König Abdulaziz Medical City (KAMC), Riad, einem akademischen Lehrkrankenhaus mit 1.500 Betten in Saudi-Arabien, zu untersuchen.

**Methoden: **Unter den Ärzt*innen in der Ausbildung am KAMC wurde zwischen Oktober und Dezember 2019 eine selbsterstellte Umfrage in Papierform verteilt. Alle Assistenzärzt*innen, die an Visiten teilnahmen, wurden zur Beteiligung aufgerufen. Der Fragebogen wurde anhand einer Umfrage erstellt, die bei einer früheren Studie verwendet wurde. Der demografische Teil erfragte Angaben zu Alter, Geschlecht, Fachrichtung und Dienstalter der Befragten. Der zweite und dritte Teil fragte nach der Logistik der gegenwärtigen Visitenpraxis. Sie umfassten verschiedene Fragen zu Struktur sowie Dauer und Häufigkeit von Visiten. Der vierte und fünfte Teil befragte die Teilnehmer*innen nach ihrer Wahrnehmung der Möglichkeiten und Hindernisse von Visiten für das Lernen. Die nachfolgenden Abschnitte stellten verschiedene Fragen zu Visitenstruktur und klinischen Ausbildern. Antworten wurden in Form einer 5-Punkte Likert-Skala erbeten (stimme überhaupt nicht zu, stimme nicht zu, unentschieden, stimme zu, stimme völlig zu). Der letzte Teil bat die Teilnehmer*innen um allgemeine Anmerkungen, Hinweise und Feedback

**Ergebnis: **Die Studie richtete sich an 250 Assistenzärzt*innen in Fachrichtungen, die routinemäßig Visiten durchführen. Nur 166 Assistenzärzt*innen reichten den Fragebogen wieder ein (Rücklaufquote von 66,4%), davon: männliche (89; 53,6%), innere (108; 65,1%), chirurgische (58; 34,9%), Assistenzärzt*innen im ersten Ausbildungsjahr (81; 48,8%). Die Gesamtdurchschnittszeit, die für Visiten aufgewendet wurde, lag bei 13 (± 11 SD) Stunden pro Woche. Die Visiten wurden als gute Möglichkeit angesehen, um etwas über diagnostische Untersuchung (138; 83%) und Patientenmanagement (133; 80,1%), Anamnese (114; 68,7%), körperliche Untersuchung (103; 62,0%) und Zeitmanagementfähigkeiten (86; 51,8%) zu lernen. Die Mehrzahl unserer Ärzt*innen in der Ausbildung hatten den Eindruck, dass die Visiten für ihre Ausbildung sehr nützlich sind (86; 52%) und zu mindestens einem Drittel der Ausbildung, die sie während ihres Studiums erhalten, beitragen. Sie berichteten auch, dass etwa ein Viertel der bei Visiten verbrachten Zeit auf die Lehre verwendet wird. Gute Ausbilder*innen wurden beschrieben als engagierte Lehrer*innen (137; 82,5%), jemand, der den Lernenden Feedback gibt (135; 81%), sie nicht antreibt (139; 83,7%), mit den Lernenden kommuniziert (144; 86,7%) und ihnen beratend zur Seite steht (101; 60,8%). Die Ärzt*innen in der Ausbildung identifizierten auch einige Faktoren, die ihre Ausbildung behindern, wie z. B. Zeitmangel (130; 79%) und die Anzahl der Patient*innen (129; 78,3%).

**Schlussfolgerung: **Diese Studie identifiziert die Stärken und Schwächen von Visiten in unserer Einrichtung. Die Ergebnisse werden den Ausbildungsbetreuer*innen dabei helfen, diese Mängel und Faktoren, die die Ausbildung behindern, anzugehen und zu korrigieren.

## Introduction

Ward rounds (WR) have been integral to the process of teaching and learning medicine for over three hundred and fifty years. First described by Leyden in 1660 [1]; the WR, can be defined as a composite clinical practice used to review inpatients’ clinical care [2]. Guidance from The Royal College of Physicians of the United Kingdom, includes more than 30 recommendations for WR effectiveness [2]. A WR should review patients’ management and progress. It is the time to make decisions about further investigations, treatment options and discharge from hospital. The WR also provides a vital opportunity to communicate with the patient, their relatives and other healthcare professionals [3]. The WR is clearly beneficial to patient care.

Ward rounds also offer a unique opportunity for all healthcare professionals and patients to participate in education and training at the bedsides. Grant et al., 1989, reported that 58% of Senior House Officers’ learning occurred on WRs [4]. However, the perceived educational value of the WR seems to have declined with the passage of time. In 2011, Claridge concluded that only 18% of Foundation year 1 and 2 doctors’ education was provided during WR [5]. Despite the importance of clinical training, many WRs focus on service rather than training as a result of several challenges and restrictions [5].

Bedside teaching is amongst the most challenging tasks in clinical education [6]. The dynamic is complex; to allow effective training whilst maintaining WR workflow; teachers, learners and patients must co-operate [7]. Lack of engagement by any of these stakeholders can severely disrupt the learning environment and adversely affect patient care [7].

The clinical teacher’s role in facilitating and directing the WR is crucial [7]. Dewhurst (2010) analysed trainees’ perspectives on learning opportunities during WRs. The combination of poor time management with the perception of not being involved greatly reduce trainees’ perception of opportunities to learn during WRs [8]. Other factors like stressful environment and patient's lack of cooperation are important hindrances to the educational value of WR. 

With so many obstacles, it is not surprising that clinician’s lack interest and are not motivated to teach. Factors which encourage clinicians to focus on ‘business’ during WR rather than teaching include learners’ different educational backgrounds, disengagement of hospital staff, and the unpredictability of healthcare professionals’ workload in hospitals.

It is important to explore the educational value of WR. Few studies have investigated the quality and efficacy of WRs as a teaching tool. Furthermore, there are no data on the educational value of WR from academic hospitals in Saudi Arabia. The aim of this study was to assess this at King Abdulaziz Medical City, Ministry of National Guard Health Affairs, Riyadh, Saudi Arabia (KAMC).

## Methodology

Ethical approval for this study was provided by the institutional review board at King Abdullah International Medical Research Center, Riyadh, Saudi Arabia.

Between October and December, 2019, a cross sectional study was performed using a self-administered, paper-based survey which was distributed to trainee doctors at KAMC, a 1500 bed academic medical city. All residents who attended WRs with senior staff (i.e. board-certified or equivalent) at KAMC were invited to participate. Trainee doctors in specialties that do not conduct WRs (i.e. radiology, pathology, anesthesia) were excluded.

The questionnaire was adapted from a survey used in a previous study [5]. The demographic section requested details of the respondents age, gender, specialty and seniority (i.e. year of residency training). The second and third sections asked about the logistics of current ward round practices. It included several questions on the structure as well as the duration and frequency of ward rounds. The fourth and fifth sections asked for participant’s perception of the opportunities for, and the obstacles to, learning on ward rounds. The subsequent sections asked several questions on ward round structure and the clinical teacher. Responses were requested on a 5-point Likert-type scale (strongly disagree, disagree, neutral, agree, strongly agree). The last section asked the participant for general comments and feedback. 

The main grouping variables were derived from respondents’ demographics (e.g. gender, specialty and seniority). The main outcome variables were percentage of overall learning which occurs on WRs, Percentage of time devoted to teaching during an average WR. 

Statistical Product and Service Solutions (SPSS; version 20, IBM, USA) was used for data management and analysis. Categorical data were described as counts and percentages while interval data were described using means and standard deviations (Mean±SD). T test and ANOVA were used to compare the means of the main outcome variables. Significance was considered when P>0.05.

## Results

### Demographics

The study targeted 250 residents in specialties that routinely performed WRs. Only 166 residents returned the questionnaire (response rate 66.4%). The demographics of respondents is shown in table 1 (Tab. 1). Approximately a quarter of the participants were from Internal Medicine 42 (25.3%). Significant numbers of residents from pediatrics 31 (18.7%), General Surgery 28 (16.9%) and Obstetrics and Gynecology 22 (13.3%) responded. A few responses were received from residents in Orthopedics 8 (4.8%) and Neurology 6 (3.6%). Most responses came from first year residents (R1; 81, 48%). Second (R2; 28, 16.9%), third (R3; 24, 14.5%) and fourth (R4; 33, 19.9%) year residents also participated. Eighty-nine (53%) were male and 138 (83.1%) were between 25 and 29 years old (Mean 27 years±SD 2 years). The demographics of non-responders were similar.

#### Educational value of current WR

**Figure 1:**
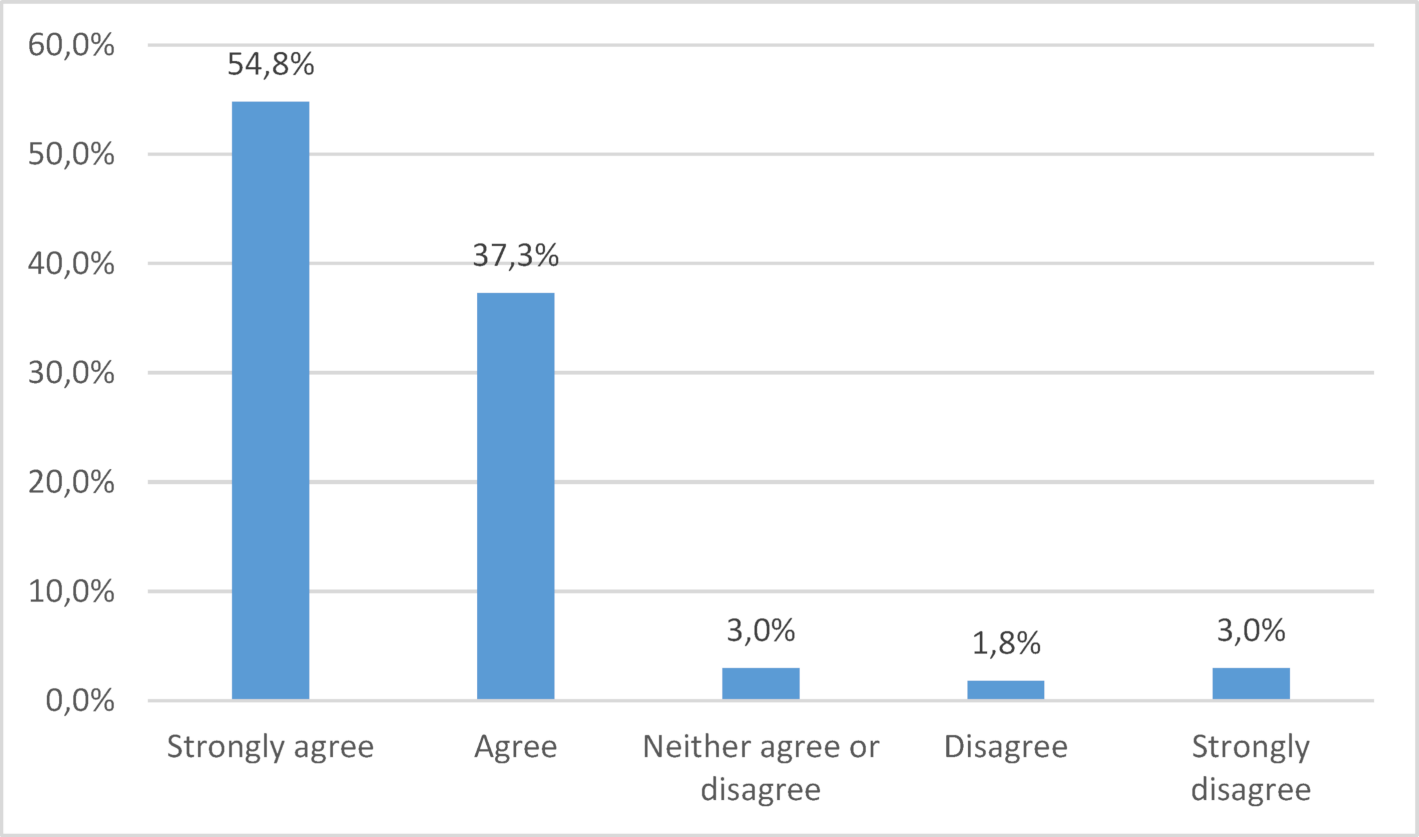
Do you agree that ward rounds could be made into a better learning experience?

Residents participated in a median of 4 (range from 1-5) senior-led WRs per week. The overall average time spent on WR was 13 (±11 SD) hours per week. While 31% (±22% SD) of participants’ learning occurs on WR, only 26% (±19% SD) of WR is devoted to teaching. The WR was perceived to be a good opportunity to learn by 52% of respondents. Figure 1 (Fig. 1) highlights participants’ perception of the educational experience of WR. The vast majority (91%) agreed or strongly agreed with the statement “WR could be made into a better learning experience” 

#### Ward rounds as a learning and teaching opportunity

Word round learning and teaching opportunity shown in table 2 (Tab. 2). Residents generally agreed that WRs were a good opportunity to learn about diagnostic investigation (83%) and patient management (80%). Fewer residents felt that WRs were a good opportunity to learn history taking (68%) and physical examination (62%).

#### Obstacles to learning and teaching on ward rounds

Factors that may negatively impact learning on WR shown in table 3 (Tab. 3). The factors which residents perceived to be the greatest barriers were; lack of time (79%), the number of patients (77%), an emphasis to get work done (66%) and a busy ward environment (57%). 

#### The effect of ward round structure on learning and teaching

While 85% of respondents said that cases should be discussed away from the bedside; only 42% reported having had the opportunity to do so. In addition, 33% of participants had experienced having questions that were not answered by the end of ward round. The morning WR was perceived to be more educationally valuable than the afternoon WR by 70% of participants. High ability to focus, better time management and more efficient workflow make the morning WR a better setting for residents’ learning. The afternoon WR is more service driven and both teachers and residents are more tired. In surgical specialties, some participants reported that the educational value of morning WR is low because of the urgency to get to the operating theatre. Other participants said that the opportunity to review patients independently before the WR increases the educational value of the WR. 

#### The effect of the teacher on the educational opportunities of ward rounds

As shown in table 4 (Tab. 4), over 80% of participants agreed or strongly agreed that, in the context of a teaching WR, the attributes of a good trainer are: enthusiasm for teaching, not being in a rush to finish, able to communicate well with the trainees and can also provide feedback. Other residents reported that a non-judgmental teacher who encourages discussion was very conducive to learning during WR. 

#### Percentage of overall learning that occurs on ward rounds and percentage of time devoted to teaching on ward rounds

Percentage of overall learning that occurs during ward rounds and percentage of time devoted to teaching during an average ward round shown in table 5 (Tab. 5). 

Participants reported that approximately 31% of their overall learning occurs during WRs (see table 5 (Tab. 5)). Approximately 25% of the time spent on WRs is devoted to teaching (see table 5 (Tab. 5)). No significant differences were found when these outcomes were stratified by gender, specialty, or seniority.

## Discussion

Assessment of trainees ‘perception of the educational value of their training program is important but is rarely done formally. 

To enhance trainee’s perception of their training and indeed improve the quality of training delivered; it is important to determine the barriers that hinder or limit learning opportunities and consider the attributes that trainees believe make a good teacher. Our study assessed these elements in one of the most pivotal workplace-based, educational activities, i.e. the WR. The crucial importance of the WR resides in its duality of purpose; patient care and staff education. Our study focused on the latter. 

The majority of our trainees felt that the WR was educationally very useful. They attributed at least a third of the education they receive during training to this single activity. Although the majority of responses were from first year residents, seniority had no significant effect on the percentage of learning that occurs during WR. 

Previous similar surveys conducted in the UK suggested a decline in the role of the WR in post-graduate training. In 1989, 58% of UK Senior House Officers’ learning was gained during WRs [4], [5]; but in 2011, WRs delivered only 18% of their training [5]. Despite the multiple sources of information and training that are available to trainees, online and offline, in classrooms and simulation labs it is reassuring that in 2019 WRs residents our institution report that WRs provide 31% of their training.

Surprisingly, there was no difference between medical and surgical specialties in the percentage of learning that occurs during WRs or the percentage of the WR time that is spent on teaching. Contrary to the caricature of surgeons rushing to finish their WR so they can “scrub in” to operate; modern surgical trainers and trainees at our institution clearly recognize the educational value of WRs for decision making and non-surgical skills.

The majority of respondents agreed or strongly agreed that the all the elements of patient management pathways are taught during WRs though to a variable extent. These include history taking, physical examination, diagnostic reasoning, interpretation of investigations, treatment and discharge planning. In the era of competency-based training, these clinical skills are not by themselves, adequate to equip trainees for their future career [9], [10]. Non-clinical skills like communication skills and time management are indispensable. 

Our study confirmed that our trainees are exposed to training in these non-technical competencies yet their satisfaction with these activities is not very high. Similarly, training in record keeping and basic sciences were not optimal. Two other negative findings were; firstly, a poor mix of service versus teaching rounds with most rounds being perceived as business rounds; and secondly, a third of trainees had unanswered questions at the end of the round. 

Our trainees reported that several serious contextual factors negatively impact the educational benefit of WRs. These are similar to findings from previous studies, examples being patient load and time constraints [5], [11], [12]. Trainer’s teaching skills and attributes are critical for effective training. Interestingly, when evaluating their perception of a good trainer, our trainees highlighted what the existing literature indicate as being essential elements [13], [14], [15]. The 5 top ranked attributes of the ten that the questionnaire included were: enthusiasm for teaching, not being in a rush, ability to communicate with the trainee and patient effectively, and giving feedback. Our trainees were happy to receive training from any board-certified or equivalent member of staff (i.e. assistant consultant, associate consultant or consultant). Although, on the whole, the findings of our study are positive, it did highlight serious misgivings. 

Over 90% of trainees were clearly unhappy and dissatisfied with training received during WRs. This highlights that their perception of their training is that it is suboptimal. Such learner-trainer discrepancies are common and well described [5], [16], [17]. Additionally, not all the essential competencies were delivered to, and acquired by the trainees to the same level. Unfortunately, training curricula have consistently, seriously underutilized WRs [18], [19]. The literature is brimming with recommendations on how to bridge this “gap”. The best examples include using a trainee-centric approach to education [20], supported by the use of generic [21], [22], [23] or competency-based checklists [24] during the WR and setting clear goals pre-WR and providing a post-WR summary with feedback [24] are some examples. Regardless trainees require training in the skills needed for an effective WR [25], [26], [27], [28]. Such training should be initiated during the undergraduate years and reinforced early-on after graduation [25], [26], [27], [28].

### Limitations and future directives

The two important limitations of our study are its subjective nature and sole reliance on trainees’ perception to determine the educational value of the WR. Furthermore, the vast majority of responses were from post-graduate year 1 trainees. Our residency program starts in October. So, all trainees had just started their stated year of training at the time of the survey. At this stage the 1st year residents have minimal clinical experience and are generally keen to learn. This may have skewed our results in favour of the educational value of WRs in our training program. The residents’ promotion exams are held in August. Trainee’s perceptions of the educational value of WRs is therefore likely to change with the time of year and experience.

An objective assessment of the quality and comprehensiveness of the training delivered during a WR would provide useful data on the actual educational impact. This could be achieved through direct observation during the WR. An objective, post-WR assessment of clinicians’ teaching abilities and the skills acquired by trainees would also be valuable. 

Practical training of both trainers and trainees on the ideal WR structure and process may be beneficial. This education can be provided in a simulated environment [28]. Furthermore providing clear educational objectives, use of evidence-based WR checklists, monitoring WR processes and outcomes and incentivizing senior staff who successfully deliver high-quality WRs are the ingredients for improvement and success [20], [21], [22], [23], [24]. 

Regardless our observations suggest that training from a teacher and a patient in the hand (during a WR) may be worth at least two of each on the “net”.

## Competing interests

The authors declare that they have no competing interests. 

## Figures and Tables

**Table 1:**
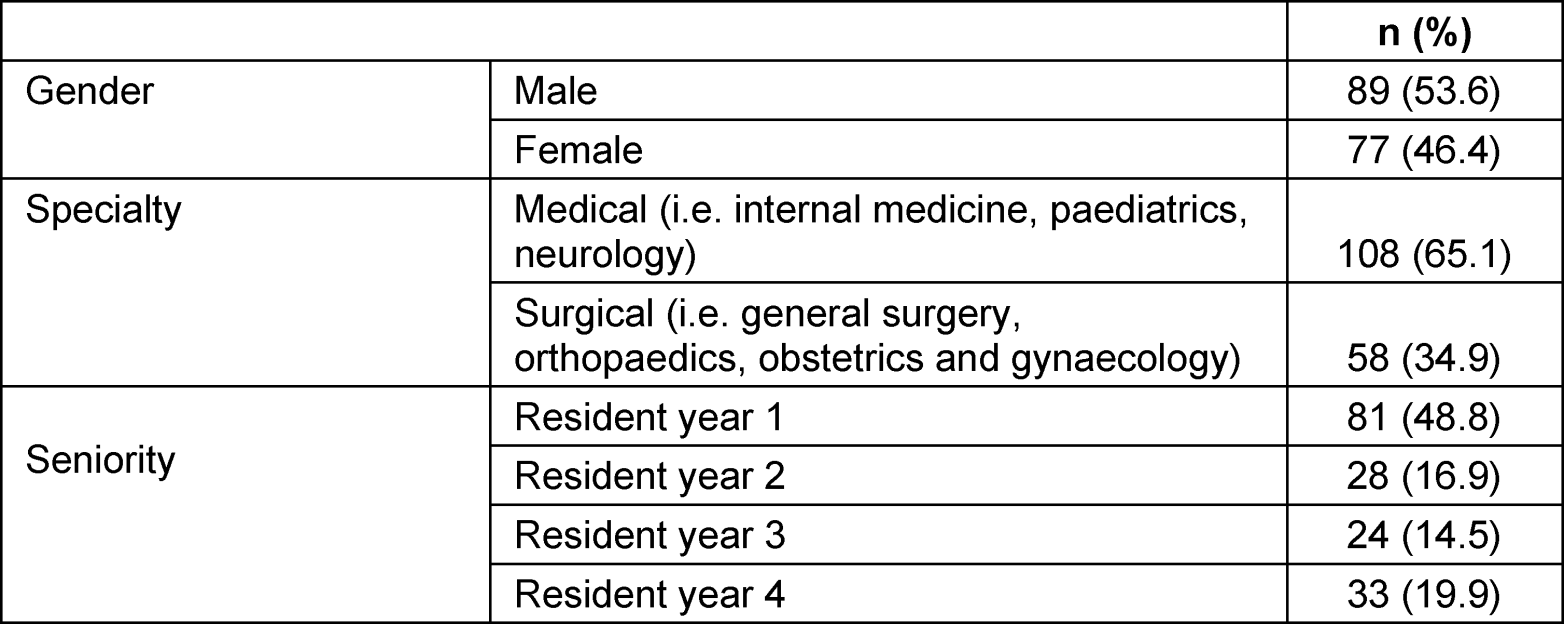
Participants demographics characteristic

**Table 2:**
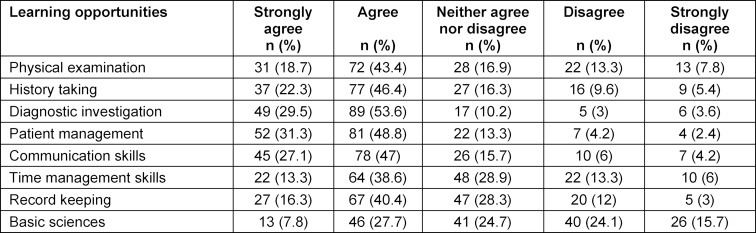
Learning opportunities on ward rounds

**Table 3:**
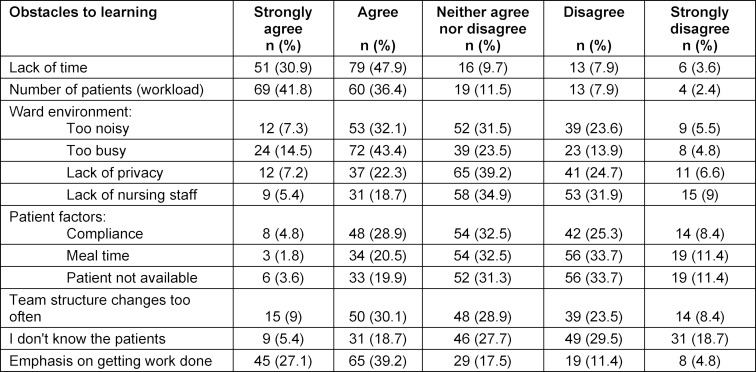
Obstacles to learning and teaching on ward rounds

**Table 4:**
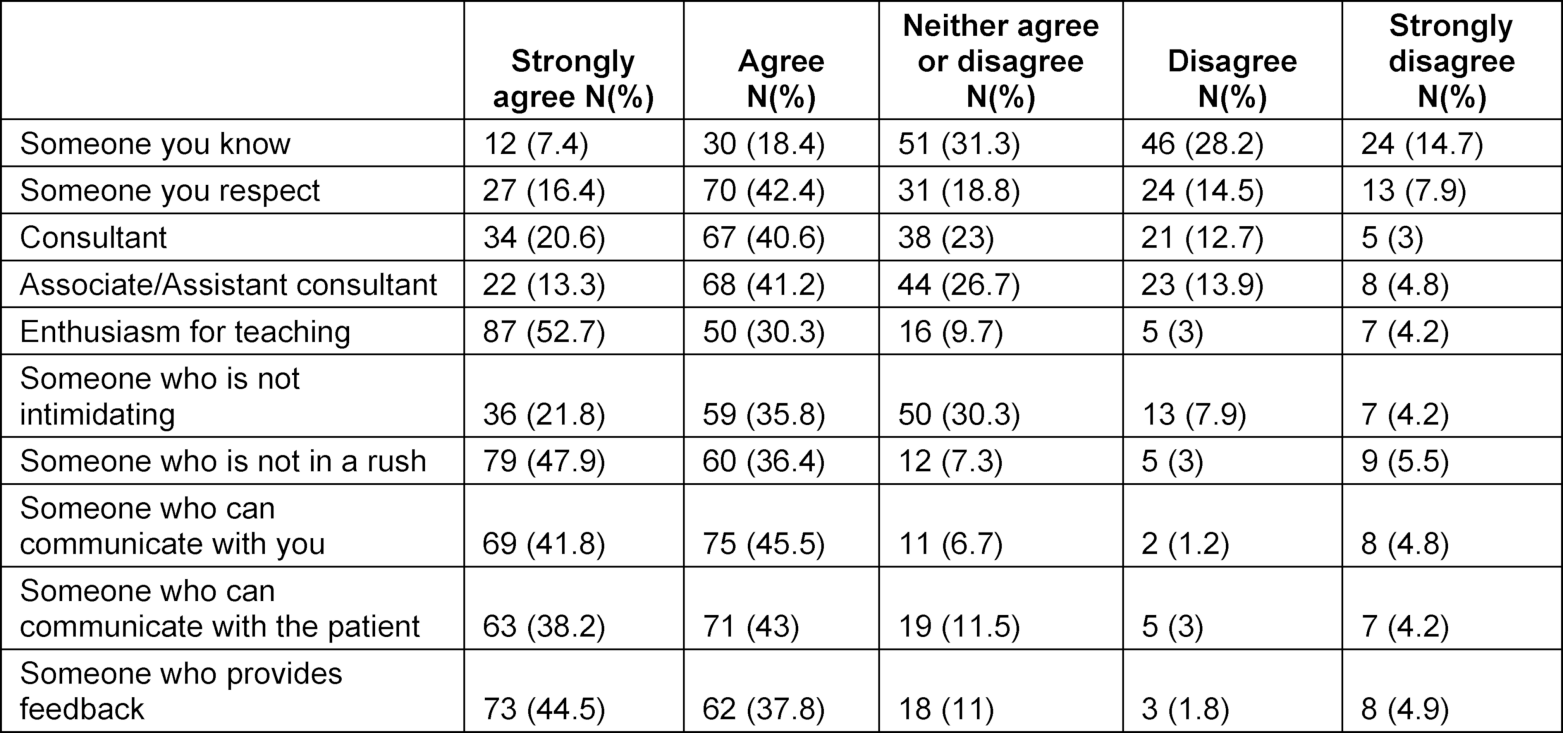
What makes a good teacher on a ward round?

**Table 5:**
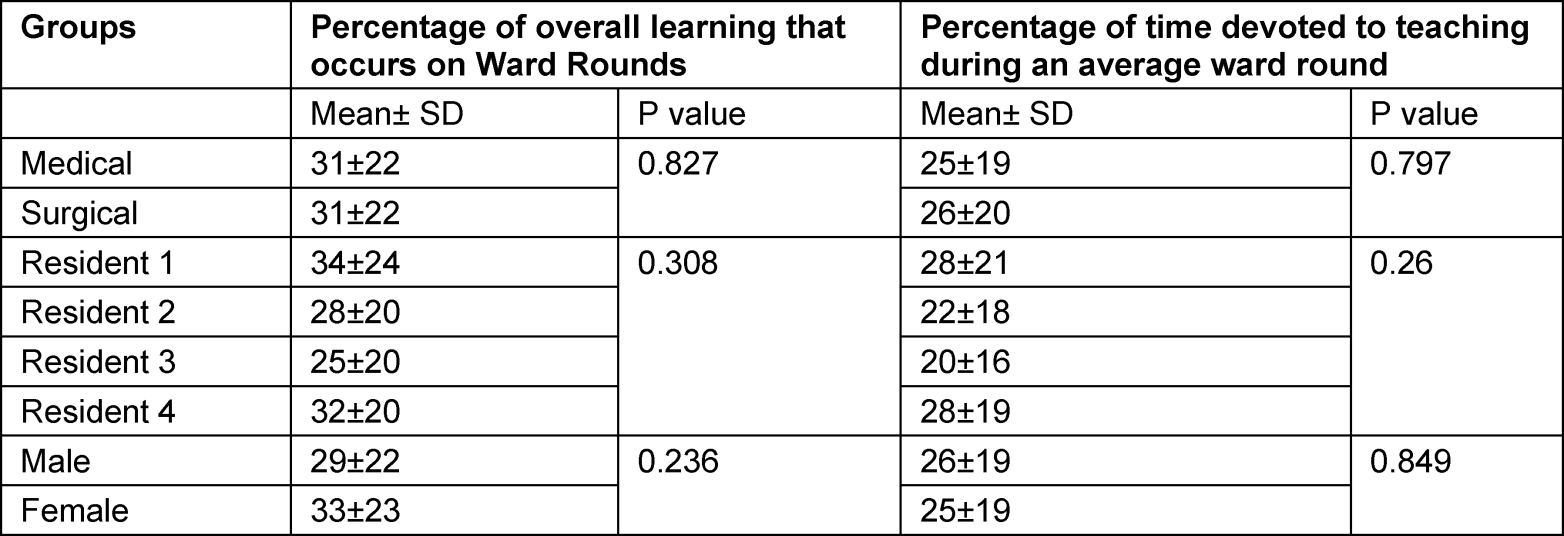
Percentage of overall learning and time devoted to teaching during an average ward round
